# High-density linkage mapping and genetic dissection of resistance to broomrape (*Orobanche crenata* Forsk.) in pea (*Pisum sativum* L.)

**DOI:** 10.3389/fpls.2023.1216297

**Published:** 2023-07-10

**Authors:** Chiara Delvento, Francesco Arcieri, Angelo Raffaele Marcotrigiano, Marzia Guerriero, Valentina Fanelli, Maria Dellino, Pasquale Luca Curci, Harro Bouwmeester, Concetta Lotti, Luigi Ricciardi, Stefano Pavan

**Affiliations:** ^1^ Department of Soil, Plant and Food Sciences, Section of Plant Genetics and Breeding, University of Bari Aldo Moro, Bari, Italy; ^2^ Institute of Biosciences and Bioresources, National Research Council (CNR), Bari, Italy; ^3^ Plant Hormone Biology Group, Swammerdam Institute for Life Sciences, University of Amsterdam, Amsterdam, Netherlands; ^4^ Department of Agricultural, Food and Environmental Sciences, University of Foggia, Foggia, Italy

**Keywords:** pea, broomrape, resistance, mapping, breeding

## Abstract

Pea (*Pisum sativum* L.) is a widely cultivated legume of major importance for global food security and agricultural sustainability. Crenate broomrape (*Orobanche crenata* Forsk.) (Oc) is a parasitic weed severely affecting legumes, including pea, in the Mediterranean Basin and the Middle East. Previously, the identification of the pea line “ROR12”, displaying resistance to Oc, was reported. Two-year field trials on a segregant population of 148 F_7_ recombinant inbred lines (RILs), originating from a cross between “ROR12” and the susceptible cultivar “Sprinter”, revealed high heritability (0.84) of the “ROR12” resistance source. Genotyping-by-sequencing (GBS) on the same RIL population allowed the construction of a high-density pea linkage map, which was compared with the pea reference genome and used for quantitative trait locus (QTL) mapping. Three QTLs associated with the response to Oc infection, named *PsOcr-1*, *PsOcr-2*, and *PsOcr-3*, were identified, with *PsOcr-1* explaining 69.3% of the genotypic variance. Evaluation of the effects of different genotypic combinations indicated additivity between *PsOcr-1* and *PsOcr-2*, and between *PsOcr-1* and *PsOcr-3*, and epistasis between *PsOcr-2* and *PsOcr-3*. Finally, three Kompetitive Allele Specific PCR (KASP) marker assays were designed on the single-nucleotide polymorphisms (SNPs) associated with the QTL significance peaks. Besides contributing to the development of pea genomic resources, this work lays the foundation for the obtainment of pea cultivars resistant to Oc and the identification of genes involved in resistance to parasitic Orobanchaceae.

## 1 Introduction

Pea (*Pisum sativum* L.) is the second most widely cultivated cool season legume in the world, yielding 12.4 Mt of dry seeds and 20.5 Mt of green production in 2021 (FAOSTAT data, 2021). In Europe, there is a positive trend towards the rise of pea cultivation, mostly related to the increased awareness of consumers on the beneficial effects of legumes on human health, the implementation of political incentives in favor of sustainable farming systems, and the growing demand for non-transgenic alternatives to soybean (Daryanto et al., 2015; Centrone et al., 2020; Costantini et al., 2021; Pavan et al., 2022).

Crenate broomrape (*Orobanche crenata* Forsk.) (Oc) is an obligate root parasite, which occurs widespread in the Mediterranean area and the Middle East and may cause up to complete yield loss on several cultivated legumes, including pea (Pavan et al., 2016; Negewo et al., 2022). The life cycle of Oc includes the stages of seed germination, attachment to the host roots, establishment of vascular connections, development of underground tubercles, emergence of floral shoots, and dispersal of seeds, which may remain viable in the soil for several decades (Xie et al., 2010; Fernández-Aparicio et al., 2016b).

Breeding for resistance proved to be a valuable strategy to cope with Oc and other parasitic weeds belonging to the botanic family of Orobanchaceae (Jamil et al., 2011; Fernández-Aparicio et al., 2014; Jamil et al., 2021; Li et al., 2023a), whereas agronomic and chemical control methods displayed limited efficacy (Fernández-Aparicio et al., 2016a). However, no Oc-resistant pea cultivar is commercially available (Rubiales, 2014; Wohor et al., 2022); thus, pea cultivation has been abandoned in several areas with a large Oc seed bank (Renna et al., 2015).

We previously reported the selection, from an Italian garden pea landrace, of the breeding line “ROR12”, displaying resistance to Oc (Pavan et al., 2016). Characterization of root extracts and exudates indicated that “ROR12” resistance might be due, at least partially, to reduced biosynthesis of strigolactones, a class of carotenoid-derived compounds acting in the rhizosphere as germination stimulants for Orobanchaceae (Yoneyama et al., 2010; Pavan et al., 2016; Bouwmeester et al., 2021). The response to Oc infection, assessed as the number of parasitic shoots emerged aboveground at crop maturity, significantly deviated from normality in an F_2_ population generated from a cross between “ROR12” and the susceptible cultivar “Sprinter”, suggesting the occurrence of one or a few loci having a major effect on the phenotype (Bardaro et al., 2016). In addition, testing a few polymorphic marker loci on resistant and susceptible F_2_ bulks revealed a significant association between response to Oc infection and a genomic region on the pea chromosome 5LG3 (Bardaro et al., 2016).

The wild pea accession “P665” was previously reported as partially resistant to Oc (Fondevilla et al., 2010). Mapping quantitative trait loci (QTLs), based on a segregant recombinant inbred line (RIL) population originating from “P665” and the susceptible cultivar “Messire”, identified four QTLs associated with the number of parasitic shoots emerged per host plant (n°br03_1, n°br03_2, n°br03_3 and n°br04), located on the pea chromosomes 2LG1, 5LG3, 3LG5, and 1LG6 (Fondevilla et al., 2010).

Genotyping-by-sequencing (GBS) is a reduced representation library sequencing strategy allowing the cost-effective identification of thousands of single-nucleotide polymorphisms (SNPs) (Elshire et al., 2011; Pavan et al., 2020). GBS was successfully used in pea to generate high-density linkage maps, in which loci associated with economically important traits were positioned (Ma et al., 2017a; Barilli et al., 2018; Guindon et al., 2019). In addition, dense linkage maps can be compared to reference genome assemblies to provide genomic context to unanchored contigs and scaffolds, resolving allelism and identifying mis-joins (Fierst, 2015; Walve et al., 2019). Currently, the genome assemblies of the cultivars “Cameor” and “ZW6” are available for the scientific community working on pea (Kreplak et al., 2019; Yang et al., 2022).

Here, we describe the use of GBS for the construction of a pea high-density linkage map, which was compared with the pea reference genome. SNP data and 2-year phenotypic observations collected on the “ROR12” x “Sprinter” RIL segregant population were used to identify and annotate genomic loci associated with Oc resistance. Finally, marker assays were designed and validated to assist selection in breeding programs.

## 2 Materials and methods

### 2.1 Plant material

The breeding line “ROR12” and the cultivar “Sprinter” were used in this study, together with a population of 148 F_7_ RILs obtained from their F_2_ progeny by single seed descent. “ROR12” was obtained from a local garden pea landrace by pure line selection (Pavan et al., 2016). Low strigolactone levels occurring in “ROR12” result in a slightly branched phenotype, which, however, does not cause a major penalty effect on the agronomic performance (Pavan et al., 2016). “Sprinter” is an old garden pea commercial cultivar previously shown to be highly susceptible to Oc (Bardaro et al., 2016; Pavan et al., 2016).

### 2.2 Phenotyping

Two field trials (sowing dates 3 October 2020 and 8 January 2021) were carried out at the experimental farm “P. Martucci” of the University of Bari (41°01′22.1″N 16°54′21.0″E) in a silty–clayey experimental field continuously cultivated with legumes, known to be highly infested by Oc. RILs were arranged according to a randomized block design with three blocks and one replication per block, with each replicate consisting of 10 plants distant 0.15 m in a single row. The blocks were placed orthogonally to a gradient of Oc infestation observed in the previous 2 years. To check for the homogeneity of the Oc seedbank distribution within blocks, five replicates of the parental cultivar “Sprinter” were randomly allocated in each block as positive control. No fertilization and irrigation were applied during the growing season. Pest and pathogen management was performed using single applications of deltamethrin and difenoconazole, whereas weed control was performed with pendimethalin in pre-emergence and manual weeding in post-emergence. Genotypic response to Oc infection was evaluated at crop maturity on 25 May 2020, and 31 May 2021, as the average number of parasitic shoots emerged aboveground per plant.

### 2.3 DNA extraction, GBS assay, and quality control

Leaf tissue samples were collected from three individuals of the parental lines and one individual of each RIL. DNA was isolated using the DNeasy Plant Mini Kit (Qiagen) according to the manufacturer’s protocol and checked for quality and concentration using agarose gel (0.8%) electrophoresis and the Qubit 3.0 fluorometer (Life Technologies). A multiplexed *Ape*KI-GBS library was prepared as described by Elshire et al. (2011) and sequenced by a paired-end approach using the Illumina Novoseq 6000 sequencing system (Elshire Group Ltd.). After demultiplexing with the Axe algorithm (Murray and Borevitz, 2018), reads were trimmed for adapter and reverse-barcode sequences using the batch_trim.pl script from github (https://github.com/Lanilen/GBS-PreProcess). Alignment to the *Pisum sativum* v1.0 reference genome (Kreplak et al., 2019) was performed using bowtie2 (Langmead and Salzberg, 2012). After pooling together alignments of the biological replicates of the parental lines, the Stacks pipeline (Catchen et al., 2013) with the biparental filtering mode was used for SNP calling. Further filtering was performed in TASSEL 5.2.31 (Bradbury et al., 2007) by selecting SNP loci showing polymorphism between the parental lines and associated with call rate >90%, minor allele frequency (MAF) >0.25, and heterozygous calls<5%, and maintaining individuals displaying heterozygosity<10%.

### 2.4 Linkage map construction and evaluation

The *mstmap* function of the ASMap R package (Taylor and Butler, 2017), implementing the Minimum Spanning Tree algorithm described by Wu et al. (2008), was used for linkage map construction. Default arguments within the function were applied, except for choosing the *p*-value threshold of 10^-11^ for clustering marker loci in linkage groups. Marker loci associated with double crossover events or displaying skewed segregation (*p*< 0.05 after the Bonferroni correction) were removed from analysis using the *drop.markers* function of ASMap. The performance of the *mstmap* function was checked by plotting the heat map of pairwise recombination fractions (RFs) between markers and their pairwise logarithm of odds (LOD) score of linkage. Graphical representation of the linkage map was obtained using the *iplotMap* function of the qtlcharts R package (Broman, 2015). The relation between the linkage map and the pea reference genome was investigated by plotting genetic vs. physical positions for each chromosome, using the ggplot2 R package (Wickham, 2016).

### 2.5 Estimation of heritability and QTL analysis

Broad-sense heritability (H^2^_B_) was estimated according to Schmidt et al. (2019), using the formula:


$${\text{H}}_{\text{B}}^{2}=\frac{{\text{σ}}_{G}^{2}}{\left({\text{σ}}_{G}^{2}+\frac{{\text{σ}}_{GY}^{2}}{n_{Y}}+\frac{\sigma_{\epsilon }^{2}}{n_{Y}\times n_{r}}\right)}$$

in which σ^2^_G_ is the genotypic variance, σ^2^_GY_ is the genotype-by-year variance, σ^2^_ϵ_ is the error variance, n_y_ is the number of years, and n_r_ is the number of replicates within each year. Restricted maximum likelihood (REML) estimates of variance components were obtained by fitting a random effect model with the *lmer* function of the lme4 R package (Bates et al., 2015), in which genotype, genotype-by-year, year, and replicates within years were set as random effects; the square root of phenotypic data was set as the dependent variable, with this transformation being necessary to correct for the right-skewness of the distribution. The model assumption of normality was assessed using the *qqnorm* function of the Stats R package (R Core Team, 2013). The *ranova* function of the lmerTest R package (Kuznetsova et al., 2017) was used to test the significance of the model random effect terms.

Mapping of QTLs was performed using linkage map data and best linear unbiased predictors (BLUPs) of RIL genotypic effects, which are widely used as alternative to phenotypic means to estimate genotypic values in QTL mapping studies (Ben Sadok et al., 2013; Allard et al., 2016; Molenaar et al., 2018; Wang et al., 2023). BLUPs were extracted by applying the *ranef* function of lme4 to the random effect model above described for H^2^_B_ estimation. The *cim* function of the Rqtl R package (Broman et al., 2003) was used to search for marker–trait associations by composite interval mapping (CIM). Arguments within the function were set to perform the Haley–Knott regression method and identify a LOD score QTL significance threshold based on a permutation test with 1,000 iterations. The *plot* function of the Stats R package (R Core Team, 2013) was applied on the output of the *cim* function to obtain graphs for chromosomal LOD scores. The percentage of the variance of RIL genotypic effects on the phenotype (PGE) explained by markers at QTL peaks was calculated with the formula:


$$\text{PGE}=100\times \left(1-10^{\left(-2\times \frac{LOD}{n}\right)}\right)$$

Confidence intervals were identified using the *lodint* function of Rqtl, based on two LOD score units drop from the QTL peak. Genes included in the QTL confidence intervals were extracted from the pea reference genome annotation general feature format (gff) file, which was downloaded from the Unité de Recherche Génomique Info (URGI) bioinformatics platform (https://urgi.versailles.inra.fr/). The BLAST tools implemented by the same platform were used (a) to search, in QTL confidence intervals, for homologs of the strigolactone biosynthetic genes reviewed by Mashiguchi et al. (2021); (b) for physical mapping of the simple sequence repeat (SSR) marker AD174 (Loridon et al., 2005), previously associated with “ROR12” resistance (Bardaro et al., 2016); and (c) for physical mapping of the carbonic anhydrase gene *Psat1g058960* that, according to the map of Carrillo et al. (2014), is closely linked to the RAPD marker OPAA19_702, in turn linked to the Oc resistance QTL n°br04 (Fondevilla et al., 2010). Search for defense response genes enrichment in QTL confidence intervals was performed by the gprofiler2 R package (Kolberg et al., 2020), using *p* = 0.05 as significance threshold and the Benjamini–Hochberg false discovery rate correction for multiple tests.

The effect of the different QTL combinations on the phenotype was investigated using the *effectplot* function of the Rqtl R package, which returned BLUP means and standard errors relative to different genotypic combinations at QTL pairs. Data were used to produce a custom plot using the ggplot2 R package (Wickham, 2016).

### 2.6 KASP marker development

Kompetitive Allele Specific PCR (KASP) assays were performed using two allele-specific forward primers, marked with the FAM and HEX fluorescence dyes, and a common reverse primer (**Table 1**). PCR reactions were performed at LGC genomics (Shanghai, China) according to standard protocols. Output fluorescence data were used to produce scatter plots, using the ggplot2 R package (Wickham, 2016).

**Table 1:** Details of the KASP assays designed on the QTLs *PsOcr-1*, *PsOcr-2*, and *PsOcr-3* identified in this study.

QTL	Target SNP	Allele-specific primer 1	Allele-specific primer 2	Common primer
*PsOcr-1*	C/G	GCAGGTTTTCTACTTCGATGACG	GCAGGTTTTCTACTTCGATGACC	GTCAATCCTTTTTGACCCTTGGACTAATT
*PsOcr-2*	T/C	TCATCCAAGTGGCTCCCTTTCATT	CATCCAAGTGGCTCCCTTTCATC	TGAAAGTGAATAGTGCAGATCCTTTGAATT
*PsOcr-3*	G/T	TCTACGATCAAATGCCGGATACC	GTTCTACGATCAAATGCCGGATACA	ATGCTGCAGCTCCCAAACTTCTCAT

## 3 Results

### 3.1 Phenotypic variation and heritability of Oc resistance

Two-year trials were carried out in an experimental field known to be severely infested by Oc, aiming to evaluate the response of a segregant population of 148 F_7_ RILs originating from a cross between “ROR12” and “Sprinter”. Scoring of the average number of parasitic shoots emerged aboveground per host plant indicated a fairly good uniformity of infestation within blocks, as relatively low dispersion around the mean was observed for five “Sprinter” replicates randomly allocated in each block (mean ± SD were 5.65 ± 0.35, 2.9 ± 0.14, and 2.95 ± 0.21 for the three blocks arranged in 2020, and 1.53 ± 0.12, 2.7 ± 0.46, and 3.4 ± 0.46 for the three blocks arranged in 2021).

**Figure 1:**
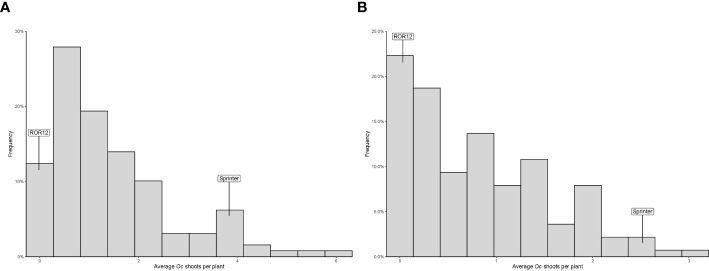
Frequency distribution of the average number of *Orobanche crenata* Forsk. (Oc) shoots emerged aboveground per host plant recorded for the parental lines “Sprinter” and “ROR12” and their RIL F_7_ population in 2020 **(A)** and 2021 **(B)**.

The average number of parasitic shoots emerged aboveground per host plant ranged, for RILs, from 0 to 6.03 in 2020, and from 0 to 3.07 in 2021. In addition, in both years, this variable exhibited a distribution clearly deviating from normality, indicating the occurrence of one or a few major loci involved in resistance (**Figure 1**). “ROR12” displayed very high resistance levels, with an average number of Oc shoots emerged per host plant of 0.13 and 0.06 in 2020 and 2021, respectively, suggesting the absence of transgressive segregation (**Figure 1**). The estimated broad-sense heritability (H^2^_B_) was 0.84, indicating a minor effect of environmental factors on phenotypic variation.

### 3.2 Construction of and evaluation of a GBS-based SNP linkage map

Sequencing of an *ApeK*I-GBS library obtained from the DNA of the parental genotypes and the RIL population resulted in approximately 2.2 million reads/sample. Approximately 33% of the reads were successfully mapped onto the pea reference genome (Kreplak et al., 2019). After the SNP call and quality control procedures, 6,182 polymorphic loci were identified. Further filtering to eliminate potentially spurious SNP calls, associated with loci displaying skewed segregation or associated with double crossover, resulted in a final panel of 4,489 markers. Of these, 4,127 were located on the seven pea chromosomes, whereas the remaining ones were located on pea superscaffolds and scaffolds. The number of polymorphic loci per chromosome and chromosome length displayed a moderate correlation (*R* ^2 ^= 0.55, *p* = 0.03), indicating a quite uniform distribution of variants across the genome.

**Figure 2:**
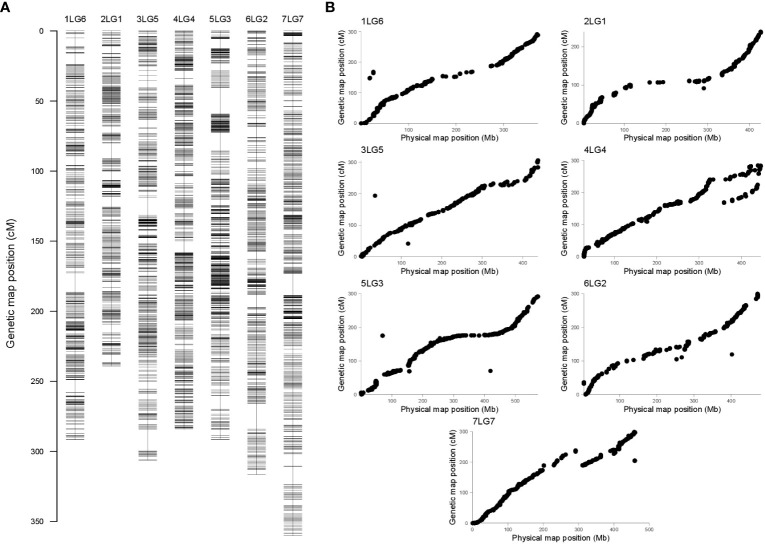
**(A)** Pea genetic map constructed with 4,489 single-nucleotide polymorphism (SNP) markers generated by genotyping-by-sequencing (GBS). Each horizontal bar indicates the marker position, expressed in centiMorgan (cM). Each linkage group (LG) has the same name of the corresponding chromosome **(B)**. Comparison between the pea genetic and physical maps. For each LG/chromosome, SNPs are represented by dots, whose coordinates on the *x* and *y* axes are given by their positions on the genetic map (cM) and the physical map (Mb), respectively.

Linkage analysis resulted in a genetic map containing seven linkage groups (LGs), in accordance with the pea haploid chromosome number (**Figure 2A**). Genetic length, physical length, and recombination rate associated with each linkage group are presented in **Table 2**. Good collinearity was found between the position of markers in the genetic map and the one in the pea reference genome (**Figure 2B**). Most notable exceptions were represented by two regions of the chromosomes 4LG4 and 7LG7 (**Figure 2B**), and the mapping of 59 markers on a different chromosome than in the reference genome (**Supplementary Table S1**). In addition, 362 SNP loci positioned on 9 superscaffolds and 125 scaffolds were anchored to the seven linkage groups (**Supplementary Table S2**).

**Table 2:** Genetic length, physical length, and recombination rate associated with the linkage groups detected in this study.

Linkage groups	Genetic length (cM)	Physical length (Mb)	Recombination rate (cM/Mb)
1LG6	291.76	371.83	0.78
2LG1	239.19	423.88	0.56
3LG5	306.22	436.66	0.70
4LG4	284.12	418.68	0.64
5LG3	291.33	572.87	0.50
6LG2	316.46	476.61	0.66
7LG7	360.01	490.63	0.73

### 3.3 Identification and annotation of three QTLs associated with response to Oc infection

**Figure 3:**
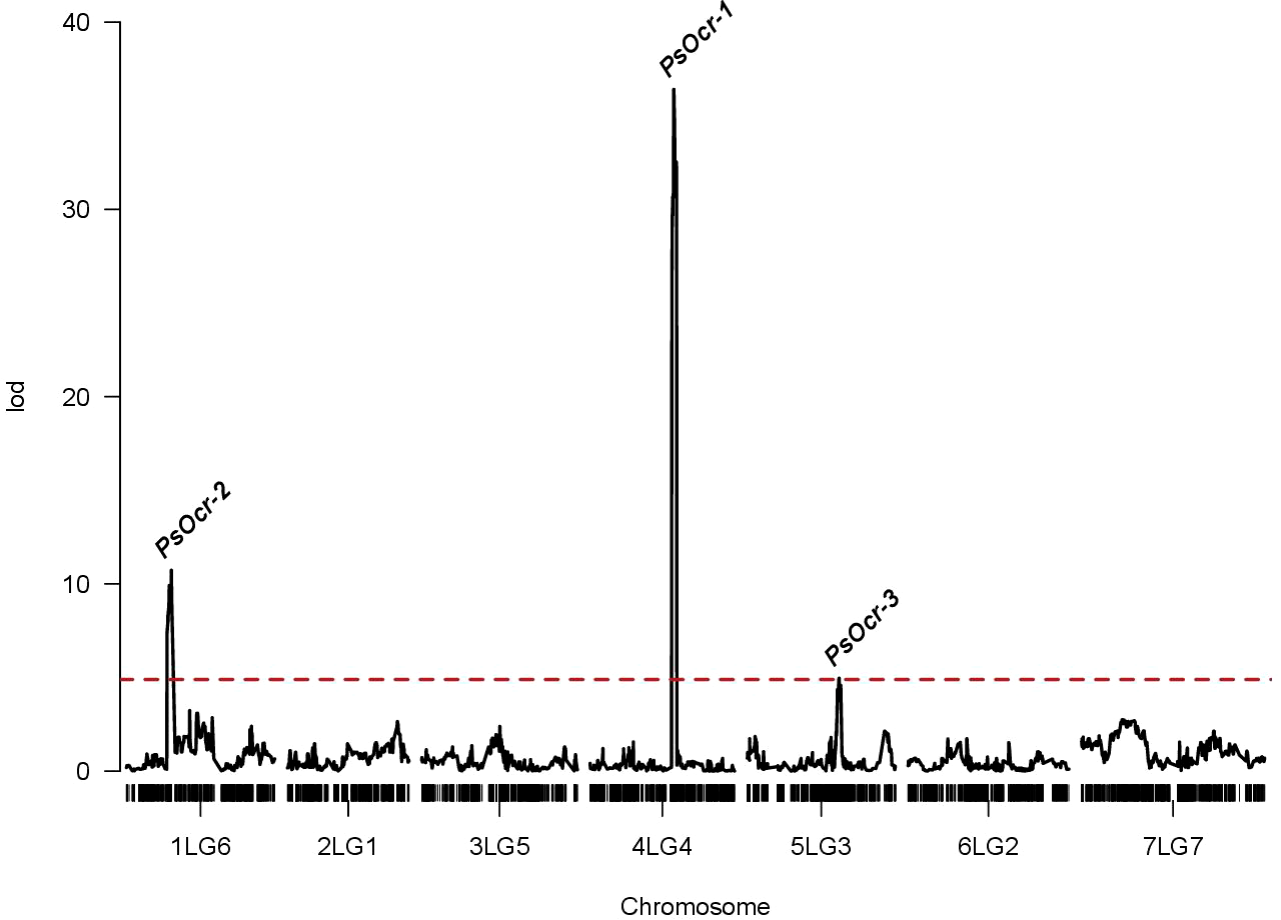
Logarithm of odds (LOD) score curves of quantitative trait loci (QTLs) associated with response to *Orobanche crenata* infection. The dashed red line indicated the significance threshold identified by permutation analysis. The three QTL above the threshold (*PsOcr-1*, *PsOcr-2* and *PsOcr-3*) are indicated in correspondence of their LOD score peaks.

Testing for the effect of variance components indicated a non-significant contribution of the genotype-by-year interaction term (**Supplementary Table S3**). Therefore, QTL mapping was performed using 2-year data, which were combined to obtain BLUPs of RIL genotypic effects on the phenotype (i.e., genotypic values). Three QTLs with LOD score peak above the significance threshold of 4.89 were identified. The QTL on chromosome 4LG4, named *PsOcr-1*, was associated with the highest LOD score peak (36.74), corresponding to 69.3% of the genotypic variance (σ^2^_G_) (**Figure 3**). The other two QTLs, located on chromosomes 1LG6 and 5LG3, were named *PsOcr-2* and *PsOcr-3*, respectively. *PsOcr-2* displayed a LOD score peak of 10.73 and explained 29.4% of σ^2^_G_, whereas *PsOcr-3* displayed a LOD score peak of 4.96 and explained 15% of σ^2^_G_. The *PsOcr-3* LOD score peak was mapped 14.98 Mb apart from the SSR marker AD174, previously associated with “ROR12” resistance (Bardaro et al., 2016). The *PsOcr-2* LOD score peak was mapped approximately 17 Mb apart from the carbonic anhydrase gene *Psat1g058960*. This gene, according to the linkage map reported by Carrillo et al. (2014), is linked to the RAPD marker OPAA19_702, which was in turn linked to the Oc resistance QTL n°br04 by Fondevilla et al. (2010).

The QTL confidence intervals spanned physical regions of 17.2 Mb for *PsOcr-1*, 7.2 Mb for *PsOcr-2*, and 51.2 Mb for *PsOcr-3*, and contained 159, 99, and 617 genes, respectively (**Supplementary Table S4**). Among these genes, 5 in *PsOcr-1* and 25 in *PsOcr-3* were annotated with the gene ontology (GO) term GO:0006952, “defense response biological process” (**Supplementary Table S4**). Enrichment analysis showed significant enrichment of *PsOcr-3* for this GO term (*p*-value = 7.08^−10^). The SNP loci corresponding to the *PsOcr-1*, *PsOcr-2*, and *PsOcr-3* LOD score peaks were positioned within genes predicted to encode an F-box domain protein (*Psat4g128720*), a phenylalanine ammonia lyase (*Psat1g046920*), and a proton-dependent oligopeptide transporter (*Psat5g221320*), respectively (**Supplementary Table S4**).

“ROR12” was previously shown to be a low-strigolactone line, which causes a reduced germination of Oc seeds (Pavan et al., 2016). Thus, we searched, within the QTL confidence intervals, for predicted genes showing homology with genes involved in the strigolactone biosynthetic pathway. This resulted in the identification, in *PsOcr-2*, of one *2-oxoglutarate-dependent dioxygenase* (*2OGD*) (*Psat1g046960*) and, in *PsOcr-3*, of one cytochrome P450 oxygenase of the CYP711A subfamily (*Psat5g201640*), two cytochrome P450 oxygenases of the CYP722C subfamily (*Psat5g209960* and *Psat5g209880*), and two *2OGD*s (*Psat5g206640* and *Psat5g206800*) (**Supplementary Table S4**).

### 3.4 QTLs for Oc resistance display both additive and epistatic effects

**Figure 4:**
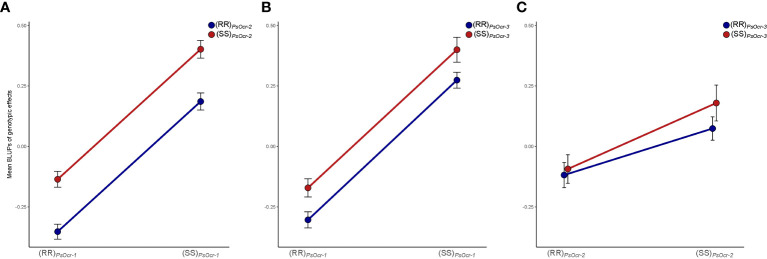
Combined effects on the phenotype of the QTL pairs *PsOcr-1*:*PsOcr-2* **(A)**, *PsOcr-1*:*PsOcr-3* **(B)**, and *PsOcr-2*:*PsOcr-3* **(C)**. Each plot reports: on the *x*-axis, the genotype for the marker locus corresponding to the first QTL LOD score peak; as a color code, the genotype for the marker locus corresponding to the second QTL LOD score peak; on the *y*-axis, mean best linear unbiased predictors (BLUPs) of genotypic effects on the phenotype. Black bars indicate ± SE of BLUP means. Lines express, for a given genotype of the second QTL, the BLUP mean change when varying the genotype at the first QTL. R and S indicate the “ROR12” and “Sprinter” alleles, respectively.

Information on the genotypic values and QTL genotypes of individual RILs was used to investigate the genetic effect of different QTL combinations. This indicated additivity between *PsOcr-1* and *PsOcr-2*, and between *PsOcr-1* and *PsOcr-3* (**Figures 4A, B**), with the three QTL alleles contributing to Oc resistance all deriving from “ROR12”. Conversely, the genotype occurring at *PsOcr-3*, homozygous for either the “ROR12” allele (R) or the “Sprinter” allele (S), did not affect the genotypic value of RILs homozygous for the R allele at *PsOcr-2* (**Figure 4C**), thus indicating epistasis of *PsOcr-2* over *PsOcr-3*.

### 3.5 Development of QTL-specific KASP markers

**Figure 5:**
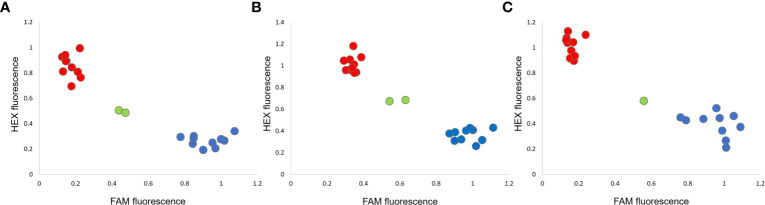
KASP assays for the *PsOcr-1* **(A)**, *PsOcr-2* **(B)**, and *PsOcr-3* **(C)** quantitative trait loci (QTLs) for resistance to *Orobanche crenata*. Each recombinant inbred line (RIL) is associated with a dot, whose coordinates are given by the fluorescence levels recorded for the FAM and HEX dyes. The red and blue colors indicate homozygous calls for the “Sprinter” and “ROR12” alleles, respectively, whereas the green color indicates heterozygous calls.

KASP assays were designed on the SNPs corresponding to the LOD score peaks of the QTLs *PsOcr-1*, *PsOcr-2*, and *PsOcr-3*. These were validated on the parental lines, as well as three different RIL panels, each one predicted from GBS data to include 10 lines homozygous for the “ROR12” allele, 10 lines homozygous for the “Sprinter” allele, and at least 1 heterozygous line. Each KASP assay yielded three fluorescence groups (**Figure 5**). In addition, KASP genotypic calls were fully consistent with GBS calls.

## 4 Discussion

Here, we show that “ROR12” resistance, highly effective against Oc, is controlled by three QTLs, including one (*PsOcr-1*) explaining as much as 69.3% of the genetic variance. In contrast, other Oc resistance sources previously identified in wild or cultivated *Pisum* germplasm confer incomplete immunity and are under the control of several minor effect QTLs (Rubiales et al., 2003; Valderrama et al., 2004; Pérez-de-Luque et al., 2005; Rubiales et al., 2005; Fondevilla et al., 2010; Rubiales et al., 2021; Wohor et al., 2022). High heritability (0.84) and the absence of pleiotropic phenotypes affecting the agronomic performance, such as severe dwarfism and extreme branching occurring in other strigolactone-defective mutants (Rameau et al., 1997; Morris et al., 2001; Pavan et al., 2016), are other features of “ROR12” resistance of value for breeding purposes. KASP technology provides a robust, high-throughput, and cost-effective solution for assisted selection (Ma et al., 2017b; Broccanello et al., 2018); therefore, the three KASP assays designed and validated in this study are expected to be of great value for the introgression of “ROR12” resistance into new pea cultivars. The KASP markers designed on the *PsOcr-2* and *PsOcr-3* LOD score peaks were mapped in proximity of the markers OPAA19_702 and AD174, suggesting that *PsOcr-2* and *PsOcr-3* might coincide with the Oc resistance QTLs previously detected by Fondevilla et al. (2010) and Bardaro et al. (2016), respectively.

The study of combined genetic effects indicated additivity between *PsOcr-1* and *PsOcr-2*, and between *PsOcr-1* and *PsOcr-3*, and epistasis of *PsOcr-2* over *PsOcr-3.* In further support of the epistasis of *PsOcr-2* over *PsOcr-3*, we found that two QTLs, *PsOcr-1* and *PsOcr-2*, are alone sufficient to explain approximately 100% of the RIL population genotypic variance. Overall, our results support the hypothesis that “ROR12” resistance originates from two independent defense mechanisms, one involving *PsOcr-1* and the other both *PsOcr-2* and *PsOcr-3*.

Several defense response genes were identified in the *PsOcr-1* and *PsOcr-3* QTL confidence intervals. Most of them encode a nucleotide-binding domain shared by apoptotic protease-activating factor-1, resistance proteins, and *Caenorhabditis elegans death-4* protein (referred to as NB-ARC domain), which was predicted to play a major role in the activation of defense responses against parasitic plants (Li and Timko, 2009; Hu et al., 2020). Interestingly, the SNP corresponding to the *PsOcr-2* LOD score peak causes a missense (Thr699Ile) mutation in the phenylalanine ammonia lyase (PAL) protein encoded by the gene *Psat1g046920*. PAL catalyzes the first committed step in the phenylpropanoid pathway, and its expression is typically induced as a defense mechanism towards biotic agents, including broomrapes (Mabrouk et al., 2016; Briache et al., 2020).

“ROR12” resistance was previously associated with reduced production of strigolactones and, consequently, reduced Oc seed germination (Pavan et al., 2016). Interestingly, the QTL confidence intervals identified in this study do not contain the two pea strigolactone biosynthetic genes characterized so far, *Rms1* and *Rms5*, encoding the Carotenoid Cleavage Dioxygenase 7 (CCD7) and Carotenoid Cleavage Dioxygenase 8 (CCD7) enzymes. However, the intervals do encompass several homologs of genes that were shown to play a role in strigolactone biosynthesis in other plant species, namely, cytochrome P450s of the CYP711A and CYP722C subfamilies and 2OGD enzymes. Members of the CYP711A subfamily play a highly important and varied role in strigolactone biosynthesis. They, for example, convert carlactone into carlactonoic acid in Arabidopsis (Abe et al. 2014), and carlactone into 4-deoxyorobanchol and 4-deoxyorobanchol into orobanchol in rice (Zhang et al., 2014). Enzymes of the CYP722C subfamily were shown to catalyze the formation of 5-deoxystrigol from carlactonoic acid in cotton (Wakabayashi et al., 2020), and of orobanchol from carlactonoic acid in tomato and cowpea (Wakabayashi et al., 2019; Wakabayashi et al., 2022). 2OGDs were associated with the biosynthesis of non-canonical strigolactones in the pea relative *Medicago truncatula* (Xie, 2016; Mashiguchi et al., 2021); however, non-canonical strigolactones have not been identified in pea. Interestingly, a recent study showed that pea is lacking a *CYP712G1* orthologue that was postulated to be required for non-canonical strigolactone biosynthesis in *M. truncatula* (Wang et al., 2022). It will be intriguing to see whether the OGDs identified in the present study play a role in the biosynthesis of so far unknown strigolactones in pea.

This study provides another successful example of mapping genes of economic interest in pea by GBS (Holdsworth et al., 2017; Gali et al., 2018; Gali et al., 2019; Pavan et al., 2022). The quality of the linkage map provided in this study is suggested by its overall collinearity with the pea reference genome (Kreplak et al., 2019). Notably, we also highlight a few exceptions to this collinearity and provide genomic context to unanchored physical scaffolds and superscaffolds, thus delivering elements for the refinement of pea genomic resources.

In conclusion, the results of this study might contribute to foster pea cultivation in the Mediterranean Basin and the Middle East, two areas in which Oc infestations discourage farmers from using legumes in crop rotations (Renna et al., 2015; Negewo et al., 2022). Future research might be addressed to the further refinement of the QTL mapping resolution. Pea is considered recalcitrant to stable genetic transformation protocols (Choudhury and Rajam, 2021); therefore, transient transformation or TILLING (Dalmais et al., 2008; Li et al., 2023b) may be used for the functional characterization of candidate genes.

## Data availability statement

The datasets presented in this study can be found in online repositories. The names of the repository/repositories and accession number(s) can be found below: https://figshare.com/, doi: 10.6084/m9.figshare.22741481.

## Author contributions

SP planned the study. LR and SP provided research funds. AM developed the RIL population. CD, FA, AM, VF, and MD contributed to data acquisition. CD, SP, MG, and PC contributed to data analysis. SP wrote the first draft of the paper. CD, PC, CL, HB, and LR critically revised the manuscript. All authors read and approved the final version of the manuscript.
